# Mitochondrial Functions Are Compromised in CD4 T Cells From ART-Controlled PLHIV

**DOI:** 10.3389/fimmu.2021.658420

**Published:** 2021-05-04

**Authors:** Juan Zhao, Madison Schank, Ling Wang, Zhengke Li, Lam Nhat Nguyen, Xindi Dang, Dechao Cao, Sushant Khanal, Lam Ngoc Thao Nguyen, Bal Krishna Chand Thakuri, Stella C. Ogbu, Zeyuan Lu, Xiao Y. Wu, Zheng D. Morrison, Mohamed El Gazzar, Ying Liu, Jinyu Zhang, Shunbin Ning, Jonathan P. Moorman, Zhi Q. Yao

**Affiliations:** ^1^ Center of Excellence in Inflammation, Infectious Disease and Immunity, James H. Quillen College of Medicine, East Tennessee State University, Johnson City, TN, United States; ^2^ Department of Internal Medicine, Division of Infectious, Inflammatory and Immunologic Diseases, Quillen College of Medicine, East Tennessee State University, Johnson City, TN, United States; ^3^ Department of Biostatistics and Epidemiology, College of Public Health, East Tennessee State University, Johnson City, TN, United States; ^4^ Hepatitis (HCV/HBV/HIV) Program, James H. Quillen VA Medical Center, Department of Veterans Affairs, Johnson City, TN, United States

**Keywords:** HIV, immune non-responder, mitochondrial dysfunction, T cell exhaustion, senescence

## Abstract

The hallmark of HIV/AIDS is a gradual depletion of CD4 T cells. Despite effective control by antiretroviral therapy (ART), a significant subgroup of people living with HIV (PLHIV) fails to achieve complete immune reconstitution, deemed as immune non-responders (INRs). The mechanisms underlying incomplete CD4 T cell recovery in PLHIV remain unclear. In this study, CD4 T cells from PLHIV were phenotyped and functionally characterized, focusing on their mitochondrial functions. The results show that while total CD4 T cells are diminished, cycling cells are expanded in PLHIV, especially in INRs. HIV-INR CD4 T cells are more activated, displaying exhausted and senescent phenotypes with compromised mitochondrial functions. Transcriptional profiling and flow cytometry analysis showed remarkable repression of mitochondrial transcription factor A (mtTFA) in CD4 T cells from PLHIV, leading to abnormal mitochondrial and T cell homeostasis. These results demonstrate a sequential cellular paradigm of T cell over-activation, proliferation, exhaustion, senescence, apoptosis, and depletion, which correlates with compromised mitochondrial functions. Therefore, reconstituting the mtTFA pathway may provide an adjunctive immunological approach to revitalizing CD4 T cells in ART-treated PLHIV, especially in INRs.

## Introduction

HIV/AIDS is characterized by a progressive depletion of CD4 T cells, leading to a gradual deficiency in host immunity, along with increased susceptibility to opportunistic infections and, ultimately, death (1). Disease progression from HIV to AIDS in untreated individuals often occurs over a period of 8-10 years, with an inexorable, virus/immune-mediated CD4 T cell destruction. Current antiretroviral therapy (ART) has markedly improved the outcome of this deadly disease by suppressing HIV replication, allowing for CD4 T cell restoration to preserve T cell numbers above the threshold (200 cells/μL) associated with immunodeficiency. However, ART does not restore all CD4 T cell subsets during chronic immune activation, thus leading to an aberrant T cell homeostasis. The dynamics of T cell homeostasis appear to result from early direct viral cytopathogenic effects, followed by an indirect effect from persistent immune activation over time in ART-controlled people living with HIV (PLHIV) (1, 2).

While ART can effectively control virus replication in the majority of PLHIV, ART does not always fully restore CD4 T cells. A substantial subgroup of PLHIV fails to recover their CD4 T cell numbers and/or functions to normal levels, and these individuals are referred to as immune non-responders (INRs) (3, 4). Even with satisfactory recovery of CD4 T cell numbers, virus-controlled subjects often exhibit both immunologic scarring and low-grade inflammation, leading to an “inflammaging” phenotype that is characterized by accelerated telomere loss, reduced proliferative capacity, low IL-2/IFN-γ production, and poor vaccine responses (5, 6). This inflammaging process exposes the immune system to unique challenges that induce T cell exhaustion and senescence, a major driver of the increased incidences of infections, cancers, cardiovascular, and neurodegenerative diseases in ART-controlled PLHIV - similar to the phenotypes often observed in the elderly (7–10). Therefore, ART-controlled, virus-suppressed HIV infection provides an excellent model for studying inflammaging in humans, and it is fundamentally important to elucidate the mechanisms underlying T cell exhaustion and senescence in PLHIV, especially in INRs.

Despite a complete control of HIV replication by ART, INRs demonstrate incomplete immune reconstitution and are at increased risk of morbidity and mortality, unlike immune responders (IRs) who have restored their CD4 T cell numbers and functions (11). While the mechanisms underlying immune failure in HIV-suppressed INRs remain elusive, several viral and host factors may play a role in this pathophysiology. In particular, ART-controlled HIV infection is characterized by the presence of viral reservoirs that prevent the eradication of HIV and possibly lead to incomplete immune reconstitution (12, 13). Also, INRs often have high levels of immune activation due to persistent, low-grade inflammation that causes inflammaging. Ultimately, HIV-INRs could result from a myriad of viral/host factors that contribute to the failure to restore CD4 T cell subsets and/or functionality. These factors may include viral proteins/RNAs/miRNAs released from HIV reservoirs, CD4 T cell nadir, age, duration of viral infection, frequent hepatitis C virus (HCV), hepatitis B virus (HBV), cytomegalovirus (CMV), Epstein–Barr virus (EBV), tuberculosis (TB), and other pathogen coinfections, cell-secreted pro-inflammatory cytokines, endogenously generated reactive oxygen species (ROS), HIV-enhanced gut permeability or altered gut microbiota, ART regimens, associated malignancies, personal stresses, and social or environmental factors (14–18). Currently, consensus criteria for defining HIV-INRs or HIV-IRs have yet to be established, but INR subjects are often characterized by significant decreases in circulating CD4 T cells (<350-500 cells/µL) (19, 20), increased frequency of cycling CD4 T cells due to immune activation, and CD4 T cells with poor responsiveness to IL-7 due to exhaustion and senescence (21, 22).

Telomere loss and mitochondrial compromise are the two most prominent features of cell aging or senescence. We have previously reported that chronic viral (HIV, HCV) infection can cause premature T cell aging, characterized by overexpression of aging markers and shortened telomeres (23–31). Since mitochondria are energy powerhouse organelles and their functions are critical for cell activity and survival (32, 33), here we analyzed CD4 T cell homeostasis, mitochondrial functions, and regulators of mitochondrial biogenesis and oxidative phosphorylation (OXPHOS) in HIV-INRs, HIV-IRs, and healthy subjects (HS). Our results revealed a contraction of the total CD4 T cell populations in HIV-INRs with a remarkable expansion of their cycling CD4 subsets and dysregulation of mitochondrial functions. Importantly, the expression of mitochondrial transcription factor A (mtTFA) was repressed in HIV-INRs, leading to compromised mitochondrial functions and aberrant T cell homeostasis. Thus, reconstituting the mtTFA pathway may provide an immunological approach for rejuvenating CD4 T cells in ART-treated PLHIV, especially in INRs.

## Methods

### Subjects

The study subjects contained 3 populations: 120 PLHIV on ART (tenofovir-based regimens, including an additional nucleoside reverse transcriptase inhibitor (NRTI),non-nucleoside reverse transcriptase inhibitor (NNRTI), and/or integrase inhibitor) with undetectable viremia (HIV-RNA <20 copies/mL), consisting of 57 HIV-IRs and 63 HIV-INRs; and 59 age-matched HS (samples supplied by BioIVT, Gray, TN) who were negative for HCV, HBV, and HIV infections. The characteristics of study subjects are shown in **Table 1**.

**Table 1:** Characteristics of the study subjects.

Subjects	*n*	Gender	Median Age	Median CD4 count
HS	59	41M/18F	45 (24-65)	N/A
HIV-IR	57	51M/6F	50 (22-69)	741 (501-1690)
HIV-INR	63	47M/16F	52 (21-71)	278 (3-491)

### Cell Isolation and Culture

Peripheral blood mononuclear cells (PBMCs) were isolated from whole blood by Ficoll density centrifugation (GE Healthcare; Piscataway, NJ). CD4^+^ T cells were isolated from PBMCs using a CD4 T Cell Isolation Kit (Miltenyi Biotec Inc; Auburn, CA) and cultured in RPMI-1640 medium containing 10% fetal bovine serum (FBS) (Atlanta Biologicals, Flowery Branch, GA), 100 IU/ml penicillin, and 2mM L-glutamine (Thermo Scientific, Logan, Utah).

### Flow Cytometry

For T cell phenotype analysis, the following fluorescence-conjugated antibodies were used: CD4-PE (Cat #300508), CD45RA-PerCP (Cat# 304156), and CD71-A647 (Cat #334118) or CD4-PerCP (Cat #300527), CD71-A647 (Cat #334118) (all from BioLegend; San Diego, CA), and CD3-PE (Cat #12-0038-42; Invitrogen; Carlsbad, CA). For intracellular staining, cells were fixed and permeabilized with the Foxp3 Transcription Factor Staining Buffer Set (Cat #00-5523-00; Invitrogen), followed by staining for mtTFA-A488 (Cat #ab198308; Abcam; Cambridge, MA) for 45 min at room temperature. For cell activation, exhaustion, senescence, and apoptosis analysis, PBMCs were thawed and stained with CD4-FITC (Cat #300506), CD71-A647 (Cat #334118), CD45RA-PerCP (Cat #304156), PD1-PE (Cat #367404), CD57-PE (Cat #322314) (all from BioLegend), or CD25-PE (Cat #12025942; eBioscience; San Diego, CA) for 30 min. For apoptosis analysis, the cells were washed twice with DPBS and stained with Annexin V-PE (Cat #BDB556422; BD Biosciences; San Jose, CA) in 1X binding buffer according to the manufacturer’s protocol. For cell proliferation, approximately 5 x 10^6^ PBMCs from HS, IRs, or INRs were labeled with 5 µM of CFSE (Cat #423801; BioLegend) and stimulated with Dynabeads (Cat #11132D; Gibco; Dublin, Ireland) at a 1:1 ratio. After 5 days, the cells were harvested and stained with CD4-PE, CD45RA-PerCP, and CD71-A647 for flow cytometry analysis. The CFSE^low^ cells were defined as undivided CD4 T cells based on the unstimulated control. For mitochondrial function analysis, the MitoTracker Green (MG; Cat #M-7514) and MitoTracker Orange (MO; Cat #M-7511; Invitrogen) probes were used according to the manufacturer’s instructions. PBMCs were thawed and cultured with 100 nM of MG or 500 nM of MO for 30 min at 37°C, then washed by DPBS and stained with CD4-PE, CD45RA-PerCP, and CD71-A647. Controls for these assays included unstained cells, isotype control antibodies, and single positive staining, which were used for gating and compensation. Samples were analyzed with a BD AccuriC6 Plus flow cytometer and FlowJo V10 software. The gating strategy is illustrated in **Supplementary Figure 1**.

### ATP Luminescence

Purified CD4 T cells were stimulated for 3 days, harvested, and plated into a 96-well plate. A standard curve was generated by preparing 8000 nM of ATP (Cat #A7699; Sigma-Aldrich; Saint Louis, MO) in complete RPMI culture medium and serial 1:1 dilution of ATP with medium. A 1:1 dilution of 100 µl CellTiter-Glo Reagent (Cat #G7570; Promega; Madison, WI) was added to all wells. Luminescence was measured by a Synergy H1 BioTek plate reader.

### Seahorse XFp Cell Mito Stress Test

Seahorse XFp Cell Mito Stress Tests (Cat# 103010-100; Seahorse, Agilent Technologies) were completed according to the manufacturer’s protocol using an XFp instrument. Briefly, CD4 T cells were purified from PBMCs, cultured in 10% FBS cRPMI with 30 IU/ml IL-2 (Cat #589104; BioLegend), and stimulated by 1μg/ml anti-CD3 (Cat # 300333) and 2 μg/ml anti-CD28 antibodies (Cat # 302943; BioLegend) for 3 days. One day before the assay, seahorse mini-cartridges were hydrated overnight in a non-CO_2_ incubator. On the day of the assay, seahorse mini plates were coated with 25 μl of 0.1 mg/ml poly-D-lysine (Cat #A3890401; ThermoFisher Scientific) for 1 h. Stimulated CD4 T cells were washed by DPBS and then plated onto pre-coated plates (2 x 10^5^/well) with Seahorse XF RPMI-1640 medium with 1.0 mM Glucose, 100 µM Pyruvate and 1.0 mM Glutamine. Data was analyzed using the Seahorse Wave software.

### Gene Microarray

Each group (HIV-INRs, HIV-IRs, and HS) consisted of isolated CD4 T cells from six subjects. Approximately 1 x 10^6^ cells from each subject were combined to form a pool of 6 x 10^6^ CD4 T cells for each study group. The gene expression analysis was performed by Arraystar Inc (Rockville, MD) and the heat map was generated using an online heatmapper software following the Average linkage clustering and Euclidean distance measurement methods (34).

### Mitochondrial DNA (mtDNA) Content and 8-Oxoguanine (8-oxoG) Measurement

mtDNA and 8-oxoG analyses were performed as described previously (24). Briefly, genomic DNA was purified from CD4 T cells stimulated with 1ug/ml anti-CD3 and anti-CD28 as described above, and the DNA concentration was measured. For mitochondrial DNA (mtDNA)/nuclear DNA (nuDNA), 25 ng of genomic DNA were used for PCR. For 8-oxoG quantification, 100 ng of DNA were treated with 10 units of Formamidopyrimidine glycosylase (Fpg, Cat# M0240L; New England Biolabs; Ipswich, MA) at 37°C for 1 h. Following digestion, 50 ng of template DNA were used for PCR.

### Western Blotting

Western blot analysis was performed as described previously (24). Primary antibodies included PGC1α (Cat #2178), mtTFA (Cat #8076), ERRα (Cat #13826), NRF-1 (Cat #46743), SOD1 (Cat #4266), GPx1 (Cat #3206), PCK1 (Cat #12940), and G6PD (Cat #12263) (all from Cell Signaling Technology; Danvers, MA), PGC1β (ab176328), PPARα (ab191226), and ACADM (ab110296) (all from Abcam). The protein bands were visualized and analyzed by the Chemi Doc Imaging System (Bio-Rad) and normalized to β-Actin (Cat# 12262; Cell Signaling Technology).

### TFAM Knockdown

CD4 T cells from HS were stimulated with dynabeads (Cat #11132D; Gibco) (at 2 cells: 1 bead ratio) for 2 days in 10% FBS cRPMI with 30 IU/ml IL-2 (Cat #589104; BioLegend). The TFAM crRNP was formed following a previously published protocol (35) and used to transfect stimulated CD4 T cells with Lonza P3 Primary Cell 4D X Kit L (Cat #V4XP-3024; Lonza; Basel, Switzerland) and program EH115, following the manufacturer’s instructions. The cells were harvested at day 3 after nucleofection for western blotting, seahorse, and mtDNA/nuDNA analysis.

### TFAM Overexpression

Purified CD4 T cells from PLHIV were stimulated with dynabeads (2 cells:1 bead ratio) for 3 days in 10% FBS cRPMI with 30 IU/ml IL-2 (Cat #589104; BioLegend). The stimulated CD4 T cells were transfected with 2.0 μg of pCMV6-AC-GFP (Cat #PS100010; OriGene; Rockville, MD) as a control or 2.0 μg of pCMV6-GFP-TFAM (Ca t#RG215488; OriGene) using a Lonza Human T Cell Nucleofector Kit (Cat #VVPA-1002; Lonza) and program T-20. Cells were collected at day 3 after transfection for western blot and seahorse mito stress test analysis.

### Statistical Analysis

The data were analyzed using Prism 7 software and are presented as mean ± SEM. The outliers were identified by the ROUT method (Q = 1.000%) and excluded from the analysis. T-tests were used to compare means of two independent groups with equal variances; Welch’s correction was utilized if unequal variances were found. Comparisons between two groups with skewed data were analyzed using the nonparametric Mann-Whitney U test. The magnitude of correlation was appropriately measured with Pearson’s correlation coefficient (parametric approach) or Spearman’s correlation coefficient (nonparametric approach) based on the property of the dataset. The correlation analyses were performed for all three groups (HS, HIV-IRs, and HIV-INRs), HS alone, and HIV (combined HIV-IRs and HIV-INRs), and the correlation coefficient (r) and p-value for the individual analyses are shown in each plot.

## Results

### Total CD4 T Cells Are Decreased While the Cycling CD4 T Cell Subset Is Increased in ART-Controlled PLHIV

**Figure 1:**
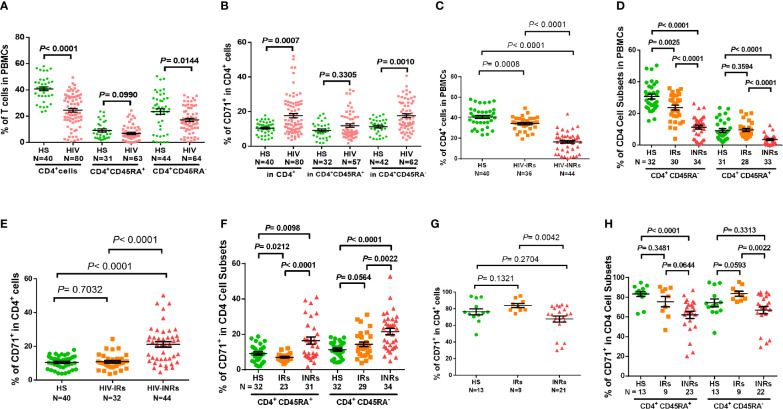
CD4 T cell homeostasis in ART-controlled PLHIV and HS. **(A, B)** Flow cytometry analysis of frequencies (%) of CD4^+^ T cell subsets within PBMCs or CD71^+^ T cells within CD4^+^ T cells isolated from ART-controlled PLHIV and HS. **(C)** Percentages of CD4^+^ T cells within PBMCs isolated from HIV-INRs, HIV-IRs, and HS. **(D)** Percentages of CD4^+^CD45RA^-^ and CD4^+^CD45RA^+^ cell subsets within PBMCs from HIV-INRs, HIV-IRs, and HS. **(E, F)** Flow cytometry analysis of CD71 expression in CD4^+^, CD4^+^CD45RA^+^, and CD4^+^CD45RA^-^ cell subsets from HIV-INRs, HIV-IRs, and HS. **(G, H)** Flow cytometry analysis of CD71 expression in CD4^+^, CD4^+^CD45RA^+^ and CD4^+^CD45RA^-^ cell subsets within PBMCs from HIV-INRs, HIV-IRs, and HS following *in vitro* TCR stimulation for 5 days.

CD4 T cell loss and/or dysfunction are the most prominent features of HIV/AIDS. To uncover the mechanisms underlying CD4 T cell homeostasis in PLHIV on ART, we analyzed the frequencies of CD4 T cell subsets in HIV-suppressed PLHIV and HS using flow cytometry. **Figure 1A** shows that the percentages of total CD4^+^ and CD4^+^CD45RA^-^ cells were significantly decreased in PBMCs from PLHIV. We then expanded our analyses using CD71 as a marker for cycling T cells, since the transferrin receptor CD71 has been identified as a surrogate for ki67 - a marker for cell proliferation (4). Interestingly, we found significantly higher frequencies of CD71^+^ cycling cells among total CD4 T cells, especially CD45RA^-^ CD4 T cells, in PLHIV compared to HS (**Figure 1B**). We also compared the frequencies of CD4 T cell subsets in HIV-INR and HIV-IR subgroups to HS. INRs and IRs were classified as having CD4 T cell counts < 500 cells/μL and > 500 cells/μL, respectively, after ART with virologic control (HIV RNA < 20 copies/mL) for at least one year. As shown in **Figure 1C**, HIV-INRs displayed a significantly lower percentage (%) of CD4^+^ T cells in PBMCs than HIV-IRs, who also showed a significantly lower frequency of CD4^+^ T cells than HS. Notably, HIV-INRs exhibited significantly decreased CD4^+^CD45RA^-^ and CD4^+^CD45RA^+^ cell populations compared to HIV-IRs and HS, whereas HIV-IRs only exhibited a significantly lower frequency of CD4^+^CD45RA^-^ (but not CD4^+^CD45RA^+^) cells compared to HS. The overall frequencies of CD4^+^CD45RA^+^ cells were much lower than CD4^+^CD45RA^-^ cells within PBMCs in all subjects (**Figure 1D**).

Counterintuitively, the total CD4 T cell population was significantly contracted within PBMCs from HIV-INRs and HIV-IRs (**Figure 1C**), while cycling CD71^+^ cells were remarkably expanded in CD4 T cells from HIV-INRs versus IRs or HS (**Figure 1E**). We did not, however, observe the same pattern in HIV-IRs vs. HS. Likewise, HIV-INRs exhibited a significant increase in CD71^+^ cycling cells in both CD4^+^CD45RA^+^ and CD4^+^CD45RA^-^ cell subsets compared to HIV-IRs and HS, whereas HIV-IRs showed an increased frequency of cycling cells only in the CD4^+^CD45RA^-^ cell subset with a significant decrease in cycling cells in CD4^+^CD45RA^+^ subset compared to HS (**Figure 1F**). HIV-INRs also showed a decrease in the frequency of CD71- noncycling cells (the major CD4 T cell population observed under unstimulated conditions) in CD4^+^CD45RA^+^ and CD4^+^CD45RA^-^ subsets compared to HIV-IRs and HS. However, the same alterations were not observed in HIV-IRs and HS (data not shown). To further characterize the cycling potential of CD4 T cells in different subjects, we stimulated the PBMCs with anti-CD3/CD28 antibodies for 5 days and examined CD71 expression. After TCR stimulation (**Figure 1G**), CD71^+^ cells became the major population within CD4 T cells, and HIV-INRs showed a relatively lower frequency of cycling CD4 T cells compared to HIV-IRs and HS. These results differed from the trend observed under the unstimulated conditions (**Figure 1E**), which showed a significant increase in the frequency of cycling CD4 T cells in HIV-INRs. Correspondingly, the same trend of low CD71^+^ cycling T cells was observed in CD4^+^CD45RA^+^ and CD4^+^CD45RA^-^ cell subsets in response to *in vitro* TCR stimulation (**Figure 1H**), suggesting a poor CD4 T cell proliferative potential in HIV-INRs compared to HIV-IRs and HS. Together, these results indicate that while total CD4 T cell numbers are diminished within PBMCs in ART-treated PLHIV, cycling cells within the CD4 subsets are expanded, especially in INRs. These findings are in line with previous observations (2–4) and support the notion of immune activation and excessive turnover of CD4^+^CD71^+^ or CD4^+^CD45RA^-^ cell subsets in ART-controlled PLHIV.

### CD4 T Cells From HIV-INRs Exhibit More Exhaustion and/or Senescence Despite ART Control of Viral Replication

We have previously shown that HIV infection drives inflammaging, during which chronic inflammation induces an immune aged phenotype, even in PLHIV on ART with undetectable viremia (23, 26). To extend this observation to different CD4 T cell subsets from our HIV subgroups, we analyzed the markers of cell activation, proliferation, exhaustion, senescence, and apoptosis in CD4 T cells from HIV-INRs, HIV-IRs, and HS. **Supplementary Figures 2A–D** shows that, while not statistically significant, HIV-INRs displayed a higher frequency of CD25 marker (also known as IL-2 receptor α chain; an early activation marker for regulatory T cell) in total CD4 T cells compared to HS and HIV-IRs, especially in CD4^+^CD71^+^ and CD4^+^CD45RA^-^ cell subsets under the unstimulated conditions, suggesting that these cells were activated *in vivo*. Importantly, the frequency of CD25^+^ cells positively correlated with the frequency of PD-1^+^ cells (an early T cell activation and exhaustion marker), but negatively correlated with the percentages of CD4 T cells in PBMCs (**Supplementary Figure S2E**), suggesting that T cell activation is associated with CD4 T cell exhaustion and depletion during HIV infection.

**Figure 2:**
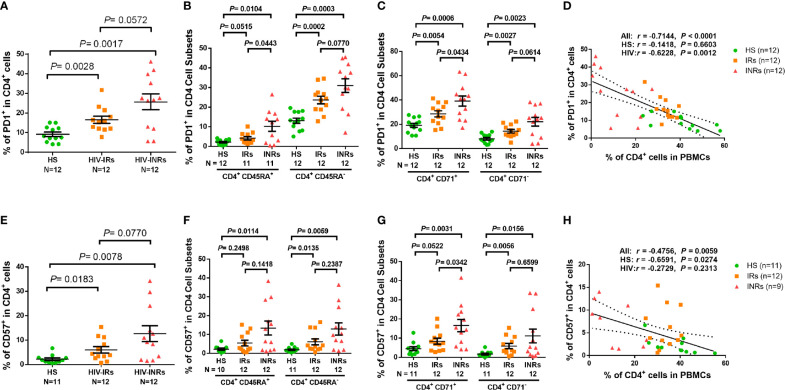
CD4 T cell exhaustion and senescence in ART-controlled PLHIV and HS. **(A–C)** Flow cytometry analysis of PD1 expression in total CD4^+^, CD4^+^CD45RA^+^, CD4^+^CD45RA^-^, CD4^+^CD71^+^, and CD4^+^ CD71^-^ cell subsets from HIV-INRs, HIV-IRs, and HS. **(D)** Spearman’s correlation between the frequency of PD1^+^ cells and the percentage of CD4^+^ T cells in HIV-INRs, HIV-IRs, and HS. **(E–G)** Flow cytometry analysis of CD57 expression in total CD4^+^, CD4^+^CD45RA^+^, CD4^+^CD45RA^-^, CD4^+^CD71^+^, and CD4^+^CD71^-^ cell subsets from HIV-INRs, HIV-IRs, and HS. **(H)** Spearman’s correlation between the frequency of CD57^+^ T cells and the percentage of CD4^+^ T cells in HIV-INRs, HIV-IRs, and HS.

Overactivation of T cells may lead to cell exhaustion. Indeed, PLHIV presented a significantly higher PD-1 expression in total CD4 T cells as well as their subsets, including CD45RA^+^, CD45RA^-^, CD71^+^ cycling, CD71^-^ non-cycling, CD71^+^CD45RA^+^, CD71^+^CD45RA^-^, CD71^-^CD45RA^+^, and CD71^-^CD45RA^-^ cells (**Supplementary Figure S2F**). PD-1 expression analysis in CD4 T cells from different HIV subgroups showed that HIV-INRs displayed an increased frequency of PD-1^+^ cells within the total CD4 population compared to HIV-IRs, who also exhibited a significantly increased percentage of PD-1^+^ cells compared to HS (**Figure 2A**). The same patterns were also observed in CD4^+^CD45RA^+^ and CD4^+^CD45RA^-^ cells (**Figure 2B**), and cycling and non-cycling CD4 T cells (**Figure 2C**), as well as their subsets (**Supplementary Figure S2G**). Overall, PD-1 expression was higher in CD4^+^CD45RA^-^ and CD4^+^CD71^+^ cell subsets compared to CD4^+^CD45RA^+^ and CD4^+^CD71^-^ cell subsets in INRs, IRs, and HS (**Figures 2B-C**). Importantly, the frequencies of PD-1^+^ cells negatively correlated with the frequencies of CD4 T cells in the peripheral blood in all subjects (**Figure 2D**).

We also analyzed the percentage of cells expressing CD57 within CD4 T cell subsets and found that PLHIV exhibited a significantly increased frequency of CD57^+^ cells in total CD4 T cells and their subsets (**Supplementary Figure S3A**). Also, HIV-INRs displayed an increased frequency of CD57^+^ cells in total CD4 (**Figure 2E**), CD4^+^CD45RA^+^ and CD4^+^CD45RA^-^ cell subsets (**Figure 2F**), and cycling and non-cycling CD4 cells (**Figure 2G**), as well as their CD4 T cell subsets compared to HIV-IRs (**Supplementary Figure 3B**), which also exhibited an increase in CD57^+^ cells compared to HS. Additionally, the frequencies of CD57^+^ cells correlated positively with the numbers of PD-1^+^ cells (**Supplementary Figure 3C**) and negatively with the percentages of CD4 T cells in PBMCs in all subjects (**Figure 2H**). Given that senescent cells are unable to undergo cell division, we were interested to measure the co-expression of CD57 (a senescence marker) and CD71 (a cycling marker) in CD4 T cells. We observed very low percentages of CD71^+^CD57^+^ cells in CD4 T cells from HS and IRs. However, the same population showed a higher frequency in CD4 T cells from INRs (**Supplementary Figure 3D**). Collectively, these results indicate that CD4 T cells from PLHIV, especially INRs, are generally more activated, exhausted, and/or senescent, despite successful control of viral replication by ART.

### CD4 T Cells From HIV-INRs Are Prone to Apoptosis and Have Poor Proliferative Capacity Despite ART Treatment

**Figure 3:**
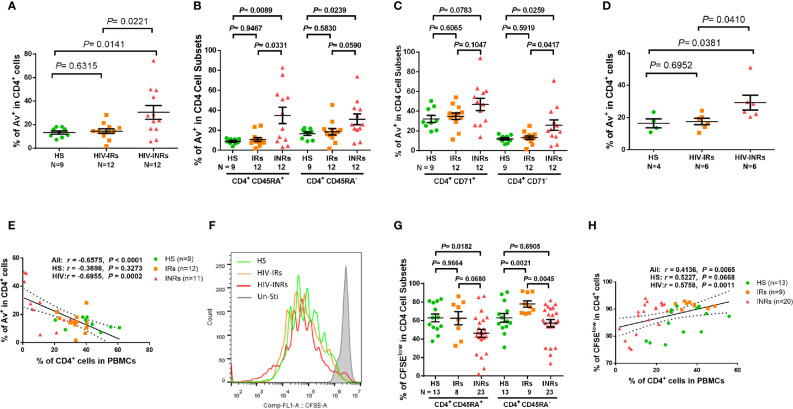
CD4 T cell apoptosis and proliferation in ART-controlled PLHIV and HS. **(A–C)** Flow cytometry analysis of Av in total CD4^+^, CD4^+^CD45RA^+^, CD4^+^CD45RA^-^, CD4^+^CD71^+^, and CD4^+^CD71^-^ cell subsets populations in HIV-INRs, HIV-IRs, and HS. **(D)** Flow cytometry analysis of Av in CD4^+^ T cells within PBMCs from HIV-INRs, HIV-IRs, and HS following *in vitro* TCR stimulation for 5 days. **(E)** Spearman’s correlation between the frequencies of Av^+^ cells and CD4^+^ T cells in HIV-INRs, HIV-IRs, and HS. **(F)** Representative histograms showing cell proliferation in CD4^+^ T cells after *in vitro* TCR stimulation for 5 days. **(G)** Frequencies of CFSE^low^ proliferative cells in CD4^+^CD45RA^+^ and CD4^+^CD45RA^-^ subsets within PBMCs from HIV-INRs, HIV-IRs, and HS after *in vitro* TCR stimulation for 5 days. **(H)** Spearman’s correlation between the frequency of CFSE^low^ proliferating cells and CD4^+^ T cell percentage in HIV-INRs, HIV-IRs, and HS.

We next sought to determine whether these exhausted and/or senescent CD4 T cells from HIV-INRs are more apoptotic, resulting in their depletion. As we previously reported (23, 26, 27) a significantly increased Annexin V positive (Av^+^) cell frequency was observed in total CD4 T cells, but not in most of the CD4 cell subsets, from PLHIV compared to HS (**Supplementary Figure 3E**). Additionally, HIV-INRs, but not HIV-IRs, exhibited an increased frequency of Av^+^ cells in total CD4 T cells compared to HS (**Figure 3A**). The same trend was observed in CD4^+^CD45RA^+^ and CD4^+^CD45RA^-^ cell subsets (**Figure 3B**), cycling and non-cycling CD4 (**Figure 3C**), CD71^+^CD45RA^+^ and CD71^+^CD45RA^-^ cell subsets, and CD71^-^CD45RA^+^ and CD71^-^CD45RA^-^ CD4 cell subsets (**Supplementary Figure S2E**) compared to HS. Moreover, HIV-INRs had significantly increased apoptosis in total (**Figure 3A**), CD4^+^CD45RA^+^ (**Figure 3B**), non-cycling (**Figure 3C**), and CD71^+^CD45RA^+^ and CD71^-^CD45RA^+^ CD4 T cell subsets (**Supplementary Figure 3F**) compared to HIV-IRs. The increases in CD4 T cell apoptosis were also observed in TCR-stimulated PBMCs from HIV-INRs compared to HIV-IRs and HS **(** **Figure 3D**), and cell apoptosis positively correlated with the PD-1 expression level (**Supplementary Figure 3G**). There were no significant differences in cell apoptosis, however, in all CD4 T cell subsets between HIV-IRs and HS under both stimulated and unstimulated conditions. Importantly, the percentage of Av^+^ cells showed a significant negative correlation with the frequency of CD4 T cells in PBMCs (**Figure 3E**), indicating that CD4 T cell apoptosis is closely associated with T cell depletion in PLHIV.

We also measured the capacity of CD4 T cell proliferation in HIV-INRs, HIV-IRs, and HS using CFSE dilution assay and flow cytometry. **Figures 3F, G** (representative overlaid histogram and summary data) shows no proliferation of CD4 T cells without TCR stimulation (grey). After 5 days of TCR stimulation, HIV-IR CD4 T cells (orange) divided multiple times and proliferated similarly (CD4^+^CD45RA^+^ cells), or even better (CD4^+^CD45RA^-^ cells) compared to HS (green). However, HIV-INR CD4 T cells (red) displayed a reduced division index compared to HIV-IRs, while the proliferation index (**Supplementary Figures 3H, I**) showed no difference between HS and INR, indicating that the reduced proliferation of INRs was due to less cells entering cell division. CD4^+^CD45RA^+^ cells from HIV-INRs also exhibited significantly poor proliferation compared to HS, but CD4^+^CD45RA^-^ cells from HIV-INR showed only slightly lower proliferation compared to HS. The overall proliferative capacity of CD4 T cells positively correlated with the CD4 cell frequency in PBMCs, i.e., CD4 T cell count and CD57 expression in CD4 T cells was closely associated with their proliferative capacity (**Figure 3H** and **Supplementary Figure 3J**). Notably, CD4 T cell proliferation capacity, as determined by the CFSE dilution (**Figure 3G**), aligned perfectly with the CD71 expression levels in response to TCR stimulation (**Figure1G, H**). Also, the frequency of CD71^+^ cycling CD4 T cells was significantly increased from 10-20% (**Figure 1E** **)** to 70-80% (**Figure 1G**) after 5 days of TCR stimulation, and the majority of CD71^+^ cycling cells proliferated well, but there were no differences in the CFSE^low^ CD4 T cell frequency amongst the three groups examined (data not shown). Together, these results indicate that HIV-INR CD4 T cells are over-activated, exhausted, and senescent, have poor proliferative capacity, and are more prone to apoptosis, all of which lead to CD4 T cell depletion.

### Mitochondrial Functions Are Aberrantly Dysregulated in CD4 T Cell Subsets in Art-Controlled PLHIV

Since mitochondria critically affect cell viability and activities (32, 33), we next examined the critical mitochondrial functions in CD4 T cells from ART-controlled PLHIV by measuring MG for mitochondrial mass, MO for mitochondrial oxidation, mtDNA/nuDNA for mtDNA copy number, oxygen consumption rate (OCR) for cellular respiration, extracellular acidification rate (ECAR) for basal glycolysis, and mitochondrial ATP production for energy power assessment.

MG selectively binds to the free thiol group of cysteine residues enriched in mitochondrial proteins regardless of the mitochondrial membrane potential, and it is commonly used as a marker for mitochondrial density. To assess mitochondrial mass, we measured MG in CD4 T cells from PLHIV and HS by flow cytometry. **Supplementary Figure 4A** shows that the median fluorescence intensity (MFI) of MG staining was slightly lower in almost all CD4 T cell subsets from HIV subjects, with a significant decrease only in CD4^+^CD71^+^CD45RA^+^ cell subsets from PLHIV compared to HS. We also analyzed the geometric MFI (gMFI) of MG in CD4 T cells and found a significant decrease in gMFI in CD4^+^CD45RA^-^, CD4^+^CD71^-^CD45RA^+^, and CD4^+^CD71^-^CD45RA^-^ cell subsets (**Supplementary Figure 4B**). We further analyzed MG MFI in CD4 T cells from HIV-INRs, HIV-IRs, and HS, including total, CD4^+^CD45RA^+^ or CD4^+^CD45RA^-^, cycling or non-cycling, CD71^+^CD45RA^+^, CD71^+^CD45RA^-^, CD71^-^CD45RA^+^, and CD71^-^CD45RA^-^ CD4 T cell subsets, and found no significant differences among the subjects or cell subsets (**Supplementary Figures 4C–F**).

**Figure 4:**
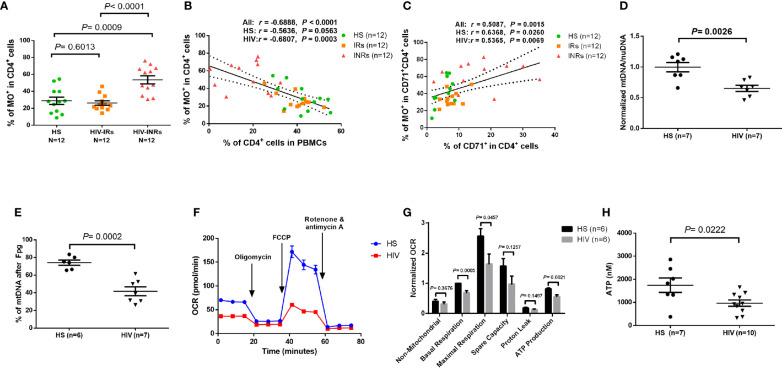
CD4 T cell mitochondrial functions in cART-controlled PLHIV and HS. **(A)** Frequency of MO^+^ cells within CD4^+^ T cells from HIV-INRs, HIV-IRs, and HS. **(B)** Pearson’s correlation between the frequencies of MO^+^ cells and CD4^+^ T cells in HIV-INRs, HIV-IRs, and HS. **(C)** Spearman’s correlation between the frequencies of MO^+^ cells in cycling CD4^+^ cells and cycling CD4^+^ T cells in HIV-INRs, HIV-IRs, and HS. **(D)** Genomic DNA were purified from stimulated CD4 T cells, followed by qPCR to determine mtDNA relative to nuDNA (normalized to HS). **(E)** Genomic DNA were purified from stimulated CD4 T cells, treated with Fpg, followed by amplification of mtDNA by qPCR. **(F, G)** Representative OCR and summary data for non-mitochondrial, basal respiration, maximal respiration, spare capacity, proton leak, and ATP production in stimulated CD4 T cells from HIV-INRs, HIV-IRs, and HS. **(H)** ATP production was measured by a CellTiter-Glo luminescent assay in stimulated CD4 T cells from PLHIV and HS.

MO stains mitochondria in live cells and accumulates in a membrane potential-dependent manner. MO is oxidized and therefore retained in actively respiring mitochondria, allowing for the assessment of mitochondrial membrane potential and oxidative phosphorylation. To assess mitochondrial oxidation, we measured MO by flow cytometry in CD4 T cells isolated from PLHIV and HS. **Supplementary Figures 4G, H** demonstrates that there were no significant differences in the MO MFI or gMFI among all CD4 T cell subsets between PLHIV and HS. However, the frequencies (%) of MO^+^ cells were increased in total CD4 T cells, and especially in the CD45RA^-^ and the CD71^-^ CD45RA^-^ CD4 T cell subsets from PLHIV compared to HS (**Supplementary Figure 4I**). We then analyzed the frequency of MO^+^ cells in all subsets of CD4 T cells from HIV-INRs, HIV-IRs, and HS. **Figure 4A** shows that HIV-INRs displayed significantly higher frequencies of MO^+^ cells than IRs and HS. Additionally, there was a very close negative correlation between the percentages (%) of the MO^+^ cells within CD4^+^ T cells and the frequencies of total CD4 T cells (**Figure 4B**), whereas the % of MO^+^ cells in cycling CD4 T cells positively correlated with the frequency of CD71^+^ cycling cells within CD4 T cells (**Figure 4C**). Also, we observed the same trend of increased frequencies of MO^+^ cells and MFI in CD4^+^CD45RA^+^, CD4^+^CD45RA^-^, cycling, non-cycling CD4 T cells, as well as all other T cell subsets in INRs compared to IRs and HS (**Supplementary Figures 5A–G**). Given that MO is a reliable readout of mitochondrial oxidation, these results indicate an aberrant mitochondrial OXPHOS and agree with our previous report showing significant increases in ROS production and apoptosis in CD4 T cells from PLHIV (23). These findings are also consistent with the decreases in total CD4 T cells (especially CD4^+^CD45RA^-^ cells) and the increases in cycling cells observed in PLHIV (particularly in INRs) (**Figure 1**), which usually occur with aberrant immune activation and excessive turnover of CD4^+^CD45RA^-^ cells during latent HIV infection.

In addition to MG and MO, we measured mitochondrial DNA content (mtDNA) and 8-oxoG (the most common oxidative DNA lesion) accumulation in mtDNA by RT-qPCR. As shown in **Figure 4D**, HIV-derived CD4 T cells exhibited significantly reduced mtDNA copy numbers relative to nuDNA contents, indicating compromised mtDNA replication and recombination, or increased mtDNA degradation and mitophagy during latent HIV infection.

Formamidopyrimidine glycosylase (Fpg) is an 8-oxoG DNA glycosylase that functions as an N-glycosylase and an apurinic (AP)-lyase. The N-glycosylase activity removes damaged purines from dsDNA, producing an AP site. The AP-lyase activity cleaves 3´ and 5´ to the AP site, thus generating a one base gap (36, 37). Therefore, the Fpg digestion of DNA can recognize and remove the accumulated 8-oxoG bases, which can be measured by PCR. To measure the most common oxidative lesion 8-oxoG levels in mtDNA, genomic DNA was purified from CD4 T cells from PLHIV and HS and then treated with mock or Fpg for 1 hour to remove 8-oxoG residues and produce a nick. Since mtDNA cleaved by Fpg cannot produce a PCR product with the selected mtDNA primers, the relative undamaged mtDNA was quantified and normalized to β2-microglobulin. As shown in **Figure 4E**, the percentage (%) of mtDNA content after Fpg digestion was significantly reduced, indicating an 8-oxoG-based accumulation of oxidative mtDNA damage during HIV infection.

We also investigated whether CD4 T cell depletion is related to abnormal mitochondrial respiration in latent HIV infection using Seahorse XFp Cell Mito Stress assays. Notably, there was no difference in OCR measurements in unstimulated CD4 T cells derived from PLHIV and HS (data not shown). **Figure 4F** shows representative OCR at basal levels and after injections of oligomycin, FCCP, and rotenone/antimycin A in stimulated CD4 T cells. Compared to HS, CD4 T cells from PLHIV exhibited significantly impaired basal and maximum mitochondrial respiratory capacity and basal glycolysis in response to anti-CD3/CD28 stimulation for 3 days (**Figure 4G** and **Supplementary Figure 5H**). Moreover, the ATP production rate was diminished in CD4 T cells from PLHIV in response to T cell receptor (TCR) stimulation, indicating a poor mitochondrial energy generation in CD4 T cells derived from PLHIV subjects compared to HS (**Figure 4G**). These results clearly reveal an impaired mitochondrial metabolic activity or poor fitness of CD4 T cells in response to TCR stimulation *in vitro*. Given the critical role of mitochondria as the energy powerhouse for cellular activities, we further measured CD4 T cell ATP production by a luminescent assay. As shown in **Figure 4H**, after *in vitro* TCR stimulation for 3 days, CD4 T cells from PLHIV displayed a much lower capacity to generate ATP compared to those from HS. These findings strongly suggest that PLHIV-CD4 T cells have abnormal mitochondrial functions - as demonstrated by the aberrant mitochondrial oxidation with oxidative stress, impaired O_2_ consumption, and decreased ATP generation - and thus are prone to apoptotic death.

### Gene Transcripts Regulating Mitochondrial Biogenesis Are Dysregulated in CD4 T Cells From PLHIV

To elucidate the mechanisms underlying the broad mitochondrial dysfunctions during HIV infection, we profiled the transcripts of genes that regulate mitochondrial biogenesis in CD4 T cells derived from PLHIV and HS using gene array analysis. Genes governing various categories of mitochondrial functions with 2-fold up- or down-regulation are listed in **Table 2**. Notably, transcriptional profiling revealed remarkable downregulation of signature genes controlling mitochondrial biogenesis and metabolism in CD4 T cells derived from HIV-INR and HIV-IR subjects compared to HS (**Supplementary Figure 6A**). The most prominent perturbations centered on the repression of those genes governing mitochondrial biogenesis (TFAM), mitochondrial OXPHOS (OXA1L, NDUFB6, ETFB, LRPPRC, SDHB, COX5B, NDUFA9, NDUFA2, MRPS12, PDHB, NDUFB4, VDAC1), oxidative defense (GPX6, SOD1, GPX7), glycolysis (ALDOA, HK1, ADH5, PDHA1, ALDH3B1), gluconeogenesis (GYS2), fatty acid and cholesterol synthesis (HMGS1, EHHADH, DGAT1, HADH, HACD3, HADHA, ACSM3, ADADSB, PPT1, ACSBG1), β-oxidation (CPT1C, CPT2, ACADM), lactate transportation (SLC16A10), and nucleotide metabolism (ADCY7) during HIV infection. Several of these genes are included in the PGC network, which is regulated by the master mitochondrial regulator PGC1α. Given the strong enrichment and importance of the PGC network genes in mitochondrial regulation, we focused our investigation on this group of genes, which showed TFAM inhibition in PLHIV, especially in INRs (**Supplementary Figure 6B**). Also, immunoblotting revealed that the majority of the PGC network genes were repressed during HIV infection, including PGC1α and its downstream signaling molecules ERRα, NRF-1, PPARα, and mtTFA in HIV CD4 T cells, especially in HIV-INR. Superoxide dismutase 1 (SOD1), a major anti-oxidative enzyme in humans, and phosphoenolpyruvate carboxykinase 1 (PCK1), a major control point for the regulation of gluconeogenesis, were also repressed in HIV-INRs, but the differences were not statistically significant in total CD4 T cells (**Supplementary Figure 6C**). Taken together, these results suggest that the critical genes governing many aspects of mitochondrial biogenesis and oxidative defense are dysregulated in CD4 T cells during HIV infection.

**Table 2:** Up- and down-regulated metabolic genes in CD4 T Cells with >2-Fold Changes.

	Category	Gene	Fold Change
**HS *vs* HIV-INR**	Glycolysis	PFKFB1	2.3835104
	Lactate production and transporters	LDHAL6A	2.0930030
		SLC16A10	-2.0675935
	Gluconeogenesis	FBP1	2.0648015
		PCK1	2.9234060
		GYS2	-2.3809115
		TKTL2	2.0310869
	Glycerol/fatty acid/cholesterol synthesis	ACACB	2.2841239
		ACSM2B	2.0050154
		ACSM3	-2.1563877
		ACSM4	2.5258243
	Serine/glycine/one-carbon metabolism	SDHAF1	2.2936909
	Fatty acid oxidation	ACADSB	-2.0406065
		SCP2	2.1914564
	Nucleotide metabolism	AMPD3	2.5768704
	Mitochondrial NADH dehydrogenase complex	NDUFB4	-2.9698385
	Mitochondrial biogenesis	TFAM	-2.3275320
**HS *vs* HIV-IR**	Glycolysis	HK1	-2.0087068
	Pentose phosphate pathway	TKTL1	2.1214849
	Glycerol/fatty acid/cholesterol synthesis	ACSM2B	3.0881022
		ACSM4	2.3388297
	Glutamine transporters and glutaminolysis	SLC1A5	2.7083647
	Fatty acid oxidation	EHHADH	2.0599924
		SCP2	2.0483894
	Nucleotide metabolism	ADCY7	-2.5875903
		AMPD3	2.2926244
		CTPS1	2.0183968
**HIV-IR *vs* HIV-INR**	Lactate production and transporters	LDHAL6A	2.7185942
	Pentose phosphate pathway	TKTL2	2.3787873
	Glycerol/fatty acid/cholesterol synthesis	ACACB	2.3868437
	Fatty acid oxidation	EHHADH	-2.4903336
	Nucleotide metabolism	IMPDH2	2.0168894
	Mitochondrial NADH dehydrogenase complex	NDUFB4	-3.1626046

### Expression of mtTFA Is Remarkably Suppressed in CD4 T Cells From PLHIV

**Figure 5:**
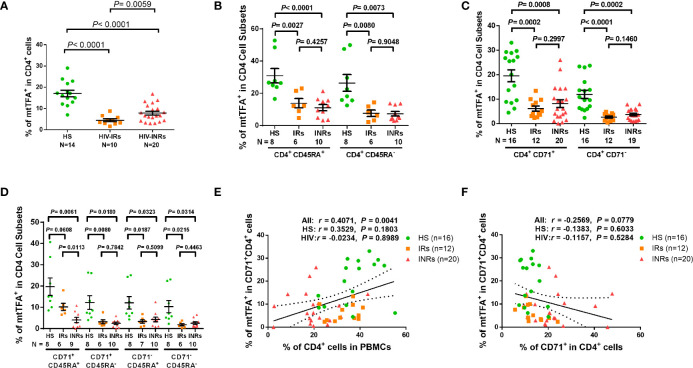
mtTFA expression in CD4 T cells in ART-controlled PLHIV and HS. **(A–D)** Flow cytometry analysis of mtTFA expression in total CD4^+^, CD4^+^CD45RA^+^, CD4^+^CD45RA^-^, CD4^+^CD71^+^, CD4^+^CD71^-^, CD4^+^CD71^+^CD45RA^+^, CD4^+^CD71^+^CD45RA^-^, CD4^+^CD71^-^CD45RA^+^, and CD4^+^CD71^-^CD45RA^+^ cell subsets from HIV-INRs, HIV-IRs, and HS. **(E)** Spearman’s correlation between the frequency of mtTFA^+^ cells in the CD71^+^CD4^+^ subset and the percentage of CD4^+^ T cells within PBMCs from HIV-INRs, HIV-IRs, and HS. **(F)** Spearman’s correlation between the frequency of mtTFA^+^ cells in the CD71^+^ CD4^+^ subset and the percentage of CD71^+^ cells in HIV-INRs, HIV-IRs, and HS.

Based on the repression of mtTFA - a downstream effector in the PGC1α network, which we have shown to be suppressed in CD4 T cells from PLHIV (24), and a key transcription factor for regulating mitochondrial genes involved in OXPHOS - we chose to examine mtTFA expression levels in order to characterize the mechanisms of compromised mitochondrial functions. We first compared the mtTFA expression in CD4 T cells from PLHIV and HS by flow cytometry. Notably, PLHIV showed a significantly lower frequency of mtTFA^+^ cells in all CD4 T cell subsets, including CD4^+^ cells and CD4^+^CD45RA^+^, CD4^+^CD45RA^-^, CD4^+^CD71^+^, CD4^+^CD71^-^, CD4^+^CD71^+^CD45RA^+^, CD4^+^CD71^+^CD45RA^-^, CD4^+^CD71^-^CD45RA^+^, and CD4^+^CD71^-^CD45RA^-^ cell subsets compared to HS (**Supplementary Figure 7A**). Also, the expression levels (MFI) of mtTFA were significantly decreased in total CD4^+^ cells and CD4+CD45RA^+^, CD4+CD45RA^-^, and CD4^+^CD71^-^ cell subsets (**Supplementary Figure 7B**). We further compared the mtTFA expression in different subsets of CD4 T cells from HIV-INRs, HIV-IRs, and HS. **Figure 5A** shows that the frequencies of mtTFA^+^ cells were significantly lower within total CD4 T cells from HIV-INRs and HIV-IRs compared to HS. A significant mtTFA inhibition was also observed in CD4^+^CD45RA^+^ and CD4^+^CD45RA^-^ cell subsets (**Figure 5B**) and cycling and non-cycling CD4 T cell subsets (**Figure 5C**) from HIV-INRs and HIV-IRs compared to HS. HIV-INRs exhibited a remarkably lower frequency of mtTFA^+^ CD71^+^CD45RA^+^ CD4 cell subset than HIV-IRs or HS, whereas both HIV-INRs and HIV-IRs showed a dramatically lower frequency of mtTFA^+^ in CD71^+^CD45RA^-^ cells as well as CD71^-^CD45RA^+^ and CD71^-^CD45RA^-^ cell subsets compared to HS (**Figure 5D**). Importantly, while the frequency of mtTFA^+^ cells within cycling CD4 T cells positively correlated with the frequency of total CD4^+^ T cells, it negatively correlated with the frequency of CD71^+^ cycling cells within the CD4 T cell population in HIV-INRs, HIV-IRs, and HS (**Figures 5E, F**). The mtTFA expression was greater in cycling than in non-cycling CD4 T cells (19.56% *v*s. 11.95% in HS), consistent with its role in regulating mitochondrial OXPHOS in these cells. We also compared the MFI of mtTFA expression in all subsets of CD4 T cells among different groups and found significant decreases in mtTFA levels in total CD4^+^, non-cycling (CD71^-^ CD4^+^), and CD45RA^+^CD4^+^ T cells in HIV-INRs compared to HS. In addition, low levels of mtTFA were detected in CD4^+^ cells and CD4^+^CD71^-^, CD4^+^CD45RA^+^, CD4^+^CD45RA^-^, and CD4^+^CD71^-^CD45RA^-^ cell subsets from HIV-IRs compared to HS (**Supplementary Figures 7C–F**). Together, these results suggest that the expression of mtTFA is significantly suppressed in most CD4 T cell subsets during HIV infection. Given its function as a master regulator of mitochondrial OXPHOS, mtTFA suppression may contribute to the compromise of mitochondrial functions, and thus disrupt CD4 T cell survival during HIV infection.

### mtTFA Controls Mitochondrial Functions *via* Regulating mtDNA During HIV Infection

**Figure 6:**
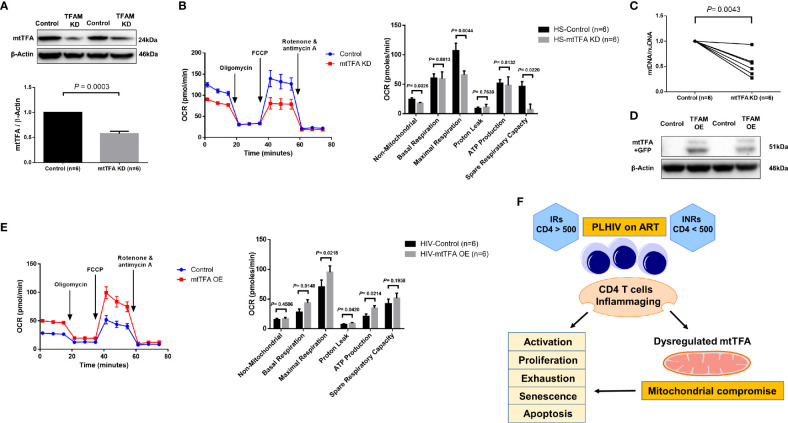
TFAM Knockdown and Overexpression in CD4 T cells from PLHIV and HS. **(A)** Western blot analysis of mtTFA and β-actin expression in HS CD4 T cells with or without TFAM KD. **(B)** Representative OCR summary data for non-mitochondrial, basal respiration, maximal respiration, spare respiration capacity, proton leak, and ATP production in HS CD4 T cells with or without TFAM KD. **(C)** Genomic DNA was isolated to determine levels of mtDNA relative to nuDNA by qPCR. DNA content from mtTFA KD was normalized to the control group. KD = knockdown. **(D)** Western blot analysis of mtTFA and β-actin expression in PLHIV CD4 T cells with or without TFAM OE. **(E)** Representative OCR summary data in CD4 T cells from PLHIV following TFAM OE. OE = overexpression. **(F)** A model depicting the mechanisms and outcomes of CD4 T cell inflammaging in HIV latency.

mtTFA is a principal mitochondrial regulator that functions by binding to mtDNA promoters responsible for regulating transcription of the mitochondrial genome (38, 39). To further elucidate the cause-effect relationship of mtTFA and its role in mitochondrial functions, we employed a novel CRISPR/Cas9 approach to knockdown (KD) mtTFA in primary CD4 T cells (35, 40). The synthesized mtTFA-crRNA/tracrRNA was delivered into TCR-activated CD4 T cells (for 48 h) from HS using a P3 Primary Cell 4D-Nucleofector X Kit. As shown in **Figure 6A**, 72 h after transfection, TFAM-KD cells exhibited a significant decrease in mtTFA protein levels as expected. Since mtTFA controls respiratory electron transport, we measured mitochondrial functions by Seahorse assay. As shown in **Figure 6B**, we observed significantly diminished maximal respiration and spare respiration in CD4 T cells after TFAM-KD. Likewise, we measured mtDNA copy numbers 3 days after TFAM-KD. As shown in **Figure 6C**, mtDNA relative to nuDNA content was significantly decreased following TFAM-KD.

To determine if overexpression of mtTFA could rescue mitochondrial functions, we ectopically expressed mtTFA in CD4 T cells derived from PLHIV. We confirmed transfection efficiency by fluorescence microscopy and flow cytometry detection of GFP-positive cells (data not shown). We further confirmed TFAM-OE by western blotting and examined mitochondrial functions by Seahorse following TFAM-OE. Western blotting clearly showed increases in mtTFA levels in TFAM-OE cells (**Figure 6D**). Seahorse OCR assay revealed rescued mitochondrial respiration, especially basal and maximal respiration (**Figure 6E**). TFAM-OE also significantly increased ATP production. Collectively, these results demonstrate that mtTFA controls mitochondrial functions and suggest that mtTFA repression plays a critical role in dampening mitochondrial fitness, and thus CD4 T cell homeostasis during HIV infection.

## Discussion

Aberrant CD4 T cell homeostasis is a major feature of HIV infection, and despite the successful suppression of viral replication by ART, a significant subgroup of PLHIV (INRs) still exhibit a phenotype of incomplete immune reconstitution, as evidenced by the failure to recover CD4 T cell numbers and/or functionality (2–4). While this failure of immunologic recovery despite potent virologic control has been well-recognized, the underlying mechanisms leading PLHIV to become INRs remain unclear. Here we employed CD4 T cells from HS and ART-controlled, virus-suppressed PLHIV (INRs and IRs) to study the role of mitochondrial dysfunctions in CD4 T cell homeostasis during latent HIV infection. We demonstrate that CD4 T cell homeostasis is disrupted in ART-controlled PLHIV and that CD4 T cells exhibit characteristics of cellular activation, exhaustion, senescence, apoptosis, and decreased proliferation and mitochondrial fitness. Based on these findings and our previous studies (23, 26, 27), we propose a model (**Figure 6F**) to illustrate the mechanisms and outcomes of CD4 T cell inflammaging during HIV latency.

In this study, we focused on PLHIV on ART because they are the major patient population in the era of ART. In this setting, how mtTFA suppression leads to compromise of the mitochondrial and CD4 T cell functions remains unclear. Due to ART control of HIV replication, and because only a very small proportion (one in a million) of PBMCs harbor HIV provirus (41), it is unlikely that HIV itself *per se* causes mtTFA suppression to compromise mitochondrial and CD4 T cell functions. We and others have shown that ART-controlled PLHIV with no viral replication can still exhibit an immune aging phenotype (7–10, 23, 26, 27). We thus believe that the mitochondrial and CD4 T cell dysregulations observed in these virus-controlled PLHIV are caused by either immunologic scarring during early active viral infection or, more likely, by low-grade inflammation during latent viral infection, or both. The CD4 T cells in PLHIV on ART exhibit an immune aging phenotype caused by a myriad of viral/host factors, including HIV reservoirs that may secrete undetectable viral components, pro-inflammatory mediators, increased ROS levels, increased gut permeability and gut microbiota, coinfection with other pathogens, ART regimens, associated malignancies, and social-related stresses, all of which may contribute to the failure to restore CD4 T cell homeostasis and/or functionality. These factors can lead to persistent, low-grade inflammation, thus driving CD4 T cell over-activation, exhaustion, senescence, apoptosis, and decreased proliferative potential and mitochondrial fitness, especially in INRs, as our results suggested. Interestingly, a recent study demonstrated differential regulation of susceptibility to HIV-1 infection in CD4 T cells based on cell activation and metabolism. Specifically, HIV-1 targets CD4 T cells with increased OXPHOS and glycolysis in an *in vitro* infection system, likely due to reduced metabolism that inhibits HIV-1 replication (42). While our data suggest that HIV infection reduces cell metabolism and fitness, it is possible that HIV preferentially infects target cells based on their metabolic status during acute infection but induces mitochondrial injuries during chronic/persistent infection, as observed in our chronically infected patients. This may be related to the differential regulation of CD4 T cell apoptosis by HIV-encoded proteins under acute versus chronic conditions, given the role of mitochondria in apoptosis (43). Alternatively, HIV-1 may differentially regulate cellular metabolism of target cells to propagate cellular infection and establish viral latency in CD4 T cells (44, 45). Particularly, this manuscript and our previous studies show that the functions of mitochondria and the integrity of chromosomal telomeres are significantly compromised during latent HIV infection - due to inhibitions of mtTFA, topoisomerase I/IIα (Top 1/2α) (26, 27), ataxia-telangiectasia mutated (ATM) (23), human telomerase reverse transcriptase (hTERT), and telomeric repeat-binding factor 2 (TRF2) (30), all of which closely correlated with CD4 T cell apoptosis and depletion, especially in INRs. Importantly, this inflammation-mediated mitochondrial compromise and telomeric DNA damage during HIV latency promote inflammaging and expose the immune system to unique challenges that could induce CD4 T cell exhaustion and senescence - a major driver of the increased incidences of infections, cancers, cardiovascular, and neurodegenerative diseases, similar to those observed in the elderly. This premature immune aging predisposes PLHIV to an increased risk of morbidity and mortality. Furthermore, key studies have shown increased activation, exhaustion, and glycolysis in CD8 T cells from PLHIV (46), suggesting that HIV infection not only targets CD4 T cell metabolism but also CD8 T cells, thus further contributing to HIV pathogenesis and immune suppression.

Mitochondria are considered the central hub of the immune system and their functions are closely related to the dynamics of cell activities (47, 48). A recent study uncovered a link between impaired T regulatory cell (Treg) proliferation and disease severity in multiple sclerosis (49). In the present study, we observed compromised mitochondrial functions *via* repression of the mtTFA pathway by which CD4 T cell homeostasis was disrupted in PLHIV, especially in INRs. This finding is consistent with a recent study showing significant repression of the PGC1α network, including mtTFA, in mice null for telomerase RNA component (TERC) or TERT genes, as disruption of the PGC network resulted in compromised mitochondrial biogenesis and functions in this model (50). Thus, it is possible that increased telomeric DNA damage and failure to repair in aging CD4 T cells in PLHIV (24) may contribute to the deregulation of the PGC network and thus mitochondrial function *via* the p53-PGC pathway (51, 52). Indeed, our gene transcription profiling and western blot data (**Table 2** and **Supplementary Figure S6**) revealed dysregulation of many target genes and key proteins in the PGC network, which regulate metabolism in CD4 T cells from PLHIV, especially in INRs. Some of these regulators are upstream of mtTFA. These deregulated genes include COX5B (cytochrome c oxidase subunit 5B); MRPS12 (mitochondrial ribosomal protein S12); NDUFA2/NDUFB4/NDUFB6/NDUFA9 (all located in mitochondrial NADH, ubiquinone dehydrogenase/oxidoreductase complex of the mitochondrial respiratory chain); VDAC1 (voltage dependent anion channel 1); and SDHB (succinate dehydrogenase complex iron sulfur subunit B). These genes also include PDHB (pyruvate dehydrogenase E1 beta subunit); ETFB (electron transfer flavoprotein subunit beta); OXA1L (a mitochondrial inner membrane protein); and LRPPRC (leucine-rich PPR motif-containing protein). Of note, mtTFA was significantly repressed in the exhausted and senescent CD4 T cells from PLHIV (**Figure 5** and **Supplementary Figure S6**). The manipulation of mtTFA revealed the link between mtTFA levels and mitochondrial functions in CD4 T cells, as demonstrated by the changes in Seahorse assay data and mtDNA contents in mtTFA-KD T cells from HS and mtTFA-OE T cells from PLHIV, respectively (**Figure 6**). Collectively, our findings uncovered the role of mtTFA repression in the deregulation of mitochondrial functions in CD4 T cells during HIV infection. Further studies to elucidate the molecular mechanisms involved in mtTFA dysregulation and its relationship with p53 and PGC1α are ongoing in our laboratory.

In summary, we have previously shown that people with chronic viral infections exhibit an immune aging phenotype, characterized by overexpression of aging markers and extremely shortened telomeres (7–10, 23, 26, 27). In the current study, we analyzed CD4 T cell homeostasis, mitochondrial functions, and regulators of mitochondrial biogenesis and OXPHOS in HIV-INRs, HIV-IRs, and HS. We demonstrated that while HIV-INRs have contracted total CD4 T cell populations, their cycling CD4 subsets are remarkably expanded and their mitochondrial functions are dysregulated. Importantly, expression of the master regulator of mitochondrial functions (mtTFA) was remarkably repressed in CD4 T cells in HIV-INRs, leading to compromised mitochondrial functions and aberrant CD4 T cell homeostasis. Thus, counteracting mtTFA repression can restore CD4 T cell homeostasis and competency in ART-treated PLHIV, especially in HIV-INRs, and thus may prevent premature immune aging. This study uncovers a pivotal molecular mechanism underlying CD4 T cell aging and informs a new approach to alleviate aberrant inflammation and avoid the consequences of immune aging associated with HIV infection.

## Data Availability Statement

The datasets generated during and/or analyzed during the current study are available from the corresponding author on reasonable request.

## Ethics Statement

All experiments involving human patients were conducted according to the ethical policies and procedures approved by the ethics committee of the joint Institutional Review Board (IRB) of East Tennessee State University and James H. Quillen VA Medical Center (ETSU/VA IRB, Approval no. 01-105s). This study was conducted in compliance with standard biosecurity and institutional safety procedures. The patients/participants provided their written informed consent to participate in this study.

## Author Contributions

JZ and MS performed most of the experiments, LW, ZKL, LNN, XD, DC, SK, LNTN, BKCT, SCO, and ZYL participated in some experiments. XYW and ZDM provided technical support. ME, JYZ, SN, and JPM offered intellectual input for troubleshooting and discussion of the findings. ZQY supervised the project and wrote the manuscript, with the help of all other authors.

## Funding

This work was supported by National Institutes of Allergy and Infectious Disease grants R01AI114748, R21AI138598, S10OD021572 (to ZQY) and 1R15AI143377 (to JPM); Veterans Affairs Merit Review Awards 1I01BX002670 and 1I01BX004281; Department of Defense Award PR170067 and ADA award 7-20-COVID-149 (to ZQY). This publication was supported with resources and facilities at the James H. Quillen Veterans Affairs Medical Center.

## Disclaimer

The contents in this publication do not represent the views of the Department of Veterans Affairs or the United States Government.

## Conflict of Interest

The authors declare that the research was conducted in the absence of any commercial or financial relationships that could be construed as a potential conflict of interest.
