# Supplementary material for: Mitochondrial Functions Are Compromised in CD4 T Cells From ART-Controlled PLHIV
Source: Front Immunol. 2021 May 4;12:658420. doi: 10.3389/fimmu.2021.658420 (PMC8129510; doi:10.3389/fimmu.2021.658420)
Supplement: Supplementary file 1 [file DataSheet_1.docx]

**Supplementary Figure Legends**

**Supplementary Figure 1. Flow cytometry gating strategy.** Representative pseudocolor plots for flow cytometry gating strategy are shown. Briefly, CD4^+^ cells were gated after gating singlets and scatter in PBMCs from HS or PLHIV. Next, the expression of CD45RA, CD71, and CD57 was analyzed in CD4^+^ cells. The expression of CD71 was further analyzed in CD4^+^CD45RA^+^ and CD4^+^CD45RA^-^ cell subsets. Finally, CD57 expression was examined in CD4^+^CD71^-^, CD4^+^CD71^+^, CD4^+^CD45RA^-^, CD4^+^CD45RA^+^, CD4^+^CD45RA^-^CD71^-^, CD4^+^CD45RA^-^CD71^+^, CD4^+^CD45RA^+^CD71^-^, and CD4^+^CD45RA^+^CD71^+^ cell subsets. Similar to CD57 staining, PBMCs were stained with anti-PD-1, Annexin V, MG, MO, or anti-mtTFA, along with anti-CD4, anti-CD45RA, and anti-CD71.

**Supplementary Figure 2. CD4 T cell activation and exhaustion in cART-controlled PLHIV and HS. A-D)** Frequencies of CD25^+^ cells within total CD4^+^ cells, CD4^+^CD71^+^, CD4^+^CD71^-^, CD4^+^CD45RA^+^, CD4^+^CD45RA^-^, CD4^+^CD71^+^CD45RA^+^, CD4^+^CD71^+^CD45RA^-^, CD4^+^CD71^-^CD45RA^+^, and CD4^+^CD71^-^CD45RA^-^ cells in HIV-INRs, HIV-IRs, and HS. **E)** Spearman’s correlation between the frequencies of CD25^+^ cells and PD1^+^ cells within the CD4 T cell population and the frequency of CD25^+^ cells and the percentage of CD4^+^ T cells in HIV-INRs, HIV-IRs, and HS. **F)** Frequencies of PD1^+^ cells within CD4^+^ cell subsets in PBMCs isolated from HIV subjects and HS. **G)** Frequencies of PD1^+^ cells within CD4^+^CD71^+^CD45RA^+^, CD4^+^CD71^+^CD45RA^-^, CD4^+^CD71^-^CD45RA^+^, and CD4^+^CD71^-^CD45RA^-^ T cell subsets from HIV-INRs, HIV-IRs, and HS.

**Supplementary Figure 3. CD4 T cell senescence, exhaustion, and apoptosis in cART-controlled PLHIV and HS. A)** Flow cytometry analysis of frequencies of CD57^+^ cells in CD4^+^ T cell subsets within PBMCs isolated from HIV subjects and HS. **B)** Frequencies of CD57^+^ cells within CD4^+^CD71^+^CD45RA^+^, CD4^+^CD71^+^CD45RA^-^, CD4^+^CD71^-^CD45RA^+^, and CD4^+^CD71^-^CD45RA^-^ T cell subsets in PBMCs from HIV-INRs, HIV-IRs, and HS. **C)** Spearman’s correlation between the frequencies of PD1^+^ cells and CD57^+^ cells within CD4^+^ T cells from HIV-INRs, HIV-IRs, and HS. **D)** Frequencies of CD57^+^CD71^+^ cells within CD4^+^ T cells from HIV-INRs, HIV-IRs, and HS. **E)** Frequencies of Av^+^ cells within CD4^+^ T cell subsets in PBMCs from HIV subjects and HS. **F)** Frequencies of Av^+^ cells within CD4^+^CD71^+^CD45RA^+^, CD4^+^CD71^+^CD45RA^-^, CD4^+^CD71^-^CD45RA^+^, and CD4^+^CD71^-^CD45RA^-^ T cell subsets within PBMCs from HIV-INRs, HIV-IRs, and HS. **G)** Spearman’s correlation between the frequencies of PD1^+^ cells and Av^+^ cells within CD4^+^ T cells from HIV-INRs, HIV-IRs, and HS. **H-I)** T cell proliferation assay. The division and proliferation indexes were calculated based on CFSE dilution in dividing cells as determined by flow cytometry. **J)** Spearman correlation between the frequency of CFSE^low^ cells and CD57^+^ cells within CD4^+^ cells.

**Supplementary Figure 4. CD4 T cell mitochondrial mass and OXPHOS in cART-controlled PLHIV and HS. A-B)** Flow cytometry analysis of MFI and gMFI of MG in CD4^+^ T cell subsets within PBMCs isolated from HIV subjects compared to HS. **C-F)** MFI of MG in total CD4^+^_,_ CD4^+^CD45RA^+^, CD4^+^CD45RA^-^, CD4^+^CD71^+^, CD4^+^CD71^-^, CD4^+^CD71^+^CD45RA^+^, CD4^+^CD71^+^CD45RA^-^, CD4^+^ CD71^-^CD45RA^+^, and CD4^+^CD71^-^CD45RA^-^ cells within PBMCs from HIV-INRs, HIV-IRs, and HS. **G-I)** MFI, gMFI, and frequency of MO in CD4^+^ T cell subsets within PBMCs from HIV subjects and HS.

**Supplementary Figure 5. CD4 T cell mitochondrial OXPHOS in cART-controlled PLHIV and HS. A-C)** Frequency of MO^+^ cells within CD4^+^CD45RA^+^, CD4^+^CD45RA^-^, CD4^+^CD71^+^, CD4^+^CD71^-^, CD4^+^CD71^+^CD45RA^+^, CD4^+^CD71^+^CD45RA^-^, CD4^+^CD71^-^CD45RA^+^, and CD4^+^CD71^-^CD45RA^-^ cell subsets within PBMCs from HIV-INRs, HIV-IRs, and HS. **D-G)** MFI of MO in total CD4^+^, CD4^+^ CD45RA^+^, CD4^+^CD45RA^-^, CD4^+^CD71^+^, CD4^+^CD71^-^, CD4^+^CD71^+^CD45RA^+^, CD4^+^CD71^+^CD45RA^-^, CD4^+^CD71^-^CD45RA^+^, and CD4^+^CD71^-^CD45RA^-^ cells within PBMCs from HIV-INRs, HIV-IRs, and HS. **H)** A summary of ECAR showing basal glycolysis in stimulated CD4 T cells from HIV-INRs, HIV-IRs, and HS.

**Supplementary Figure 6. Expression of key genes and proteins governing metabolism in CD4 T cells from cART-controlled PLHIV and HS. A)** A heat map showing the expression of genes involved in various metabolic pathways (*n*=6 per group). **B)** A representative figure with fold changes in the expression of genes involved in the PGC network (*n*=6 per group). **C)** Representative western blots and summary data of proteins involved in the PGC network (*n*=6 per group).

**Supplementary Figure 7. MtTFA expression in CD4 T cells from cART-controlled PLHIV and HS. A-B)** Frequency and MFI of mtTFA^+^ cells in CD4^+^ T cell subsets within PBMCs isolated from PLHIV and HS. **C-F)** Flow cytometry analysis of mtTFA in total CD4^+^, CD4^+^CD71^+^, CD4^+^CD71^-^, CD4^+^CD45RA^+^, CD4^+^CD45RA^-^, CD4^+^CD71^+^CD45RA^+^, CD4^+^CD71^+^CD45RA^-^, CD4^+^CD71^-^CD45RA^+^, and CD4^+^CD71^-^CD45RA^-^ cells within PBMCs from HIV-INRs, HIV-IRs, and HS.
