# Supplementary figures and images for: Mitochondrial Functions Are Compromised in CD4 T Cells From ART-Controlled PLHIV
Source: Front Immunol. 2021 May 4;12:658420. doi: 10.3389/fimmu.2021.658420 (PMC8129510; doi:10.3389/fimmu.2021.658420)

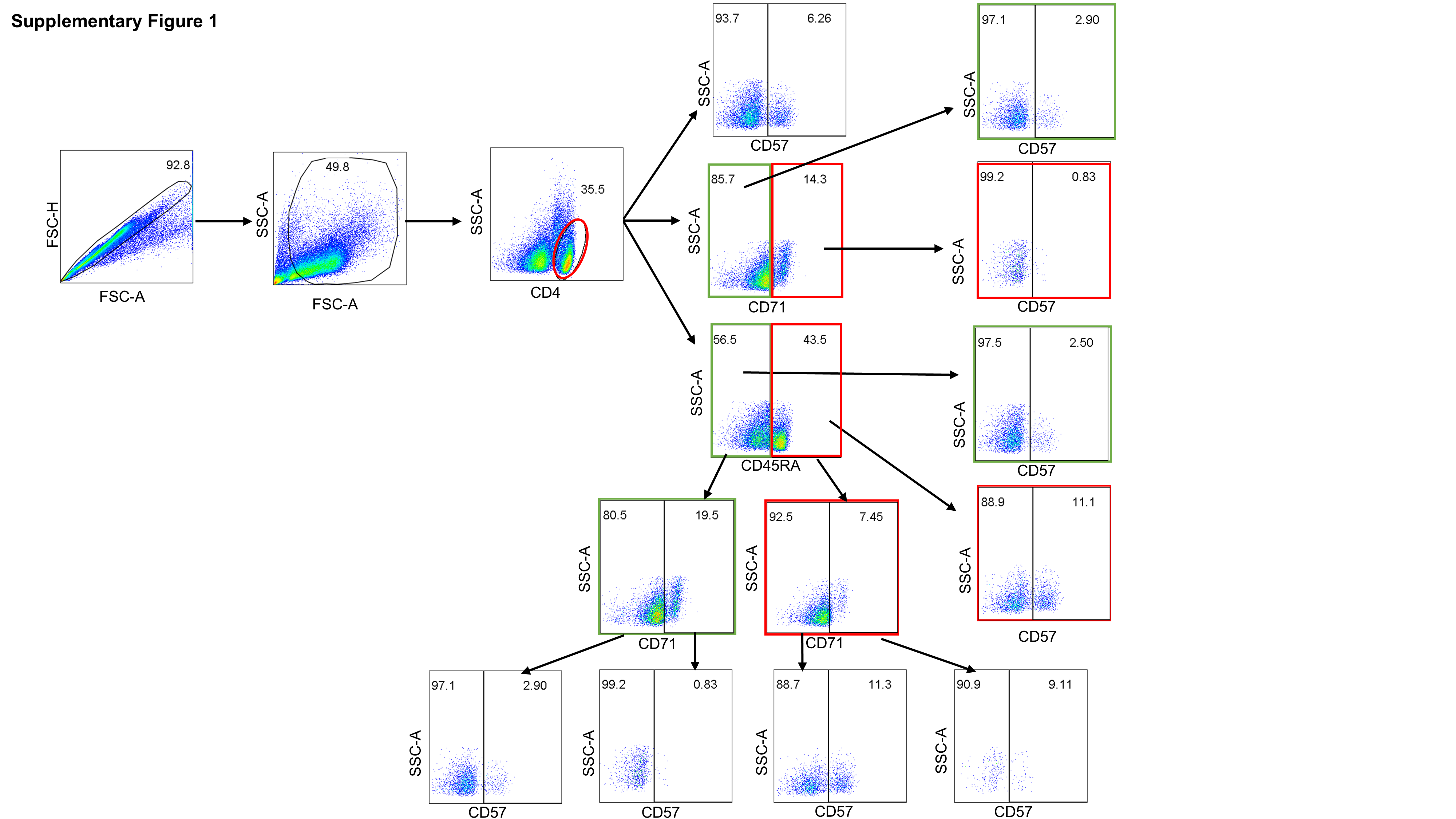

Supplement: Supplementary file 2 [file Image_1.tif]

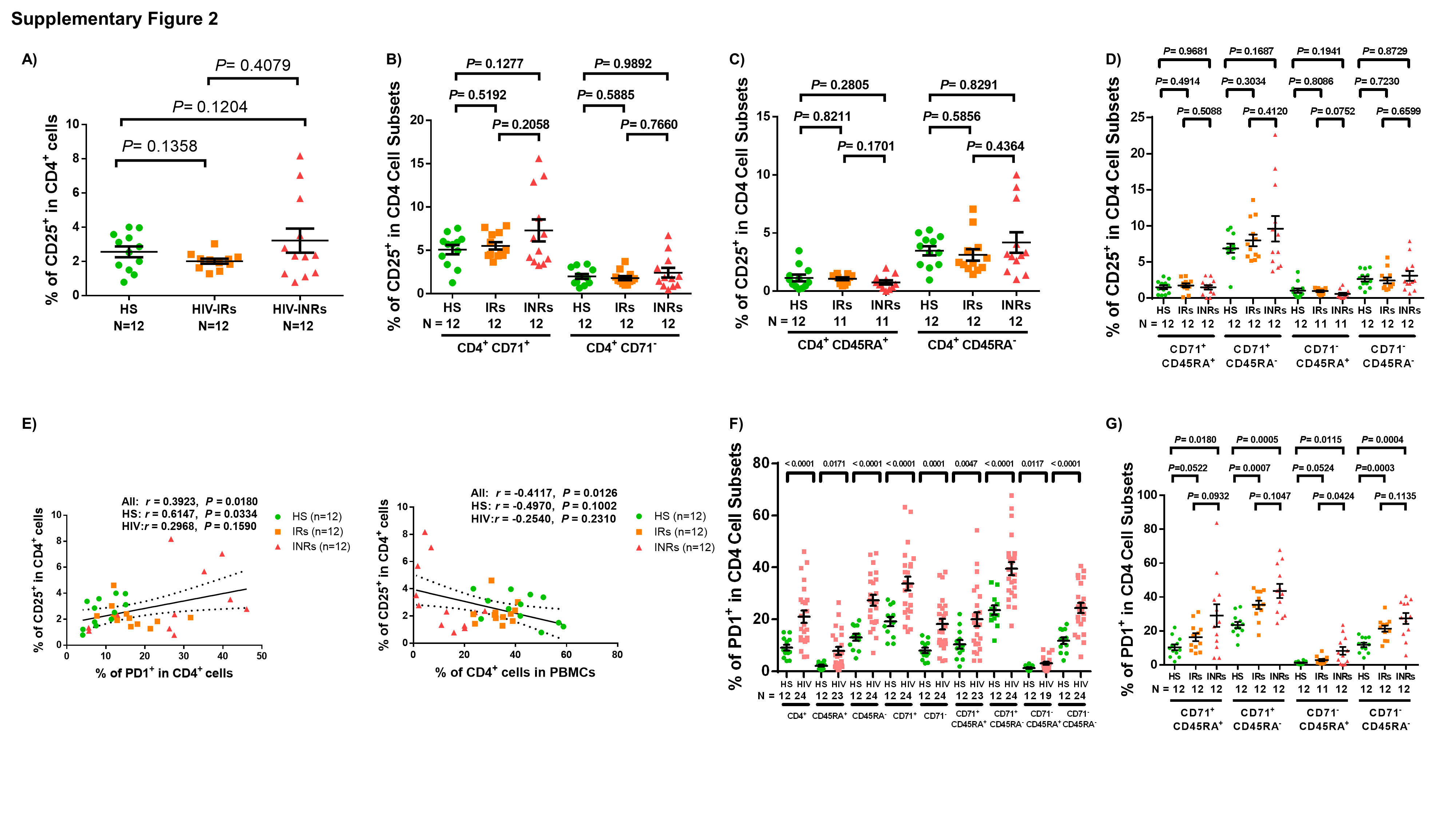

Supplement: Supplementary file 3 [file Image_2.tif]

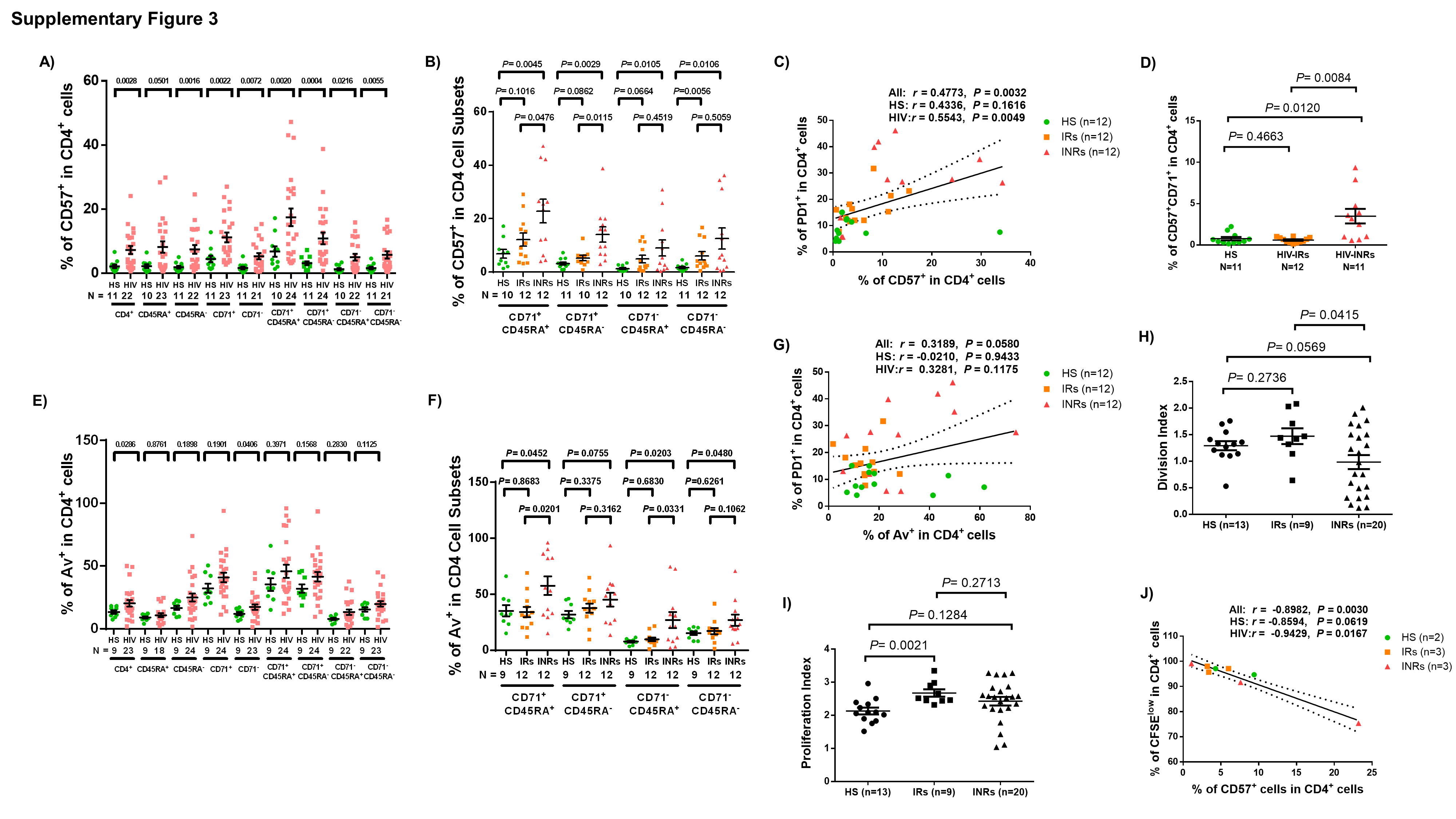

Supplement: Supplementary file 4 [file Image_3.tif]

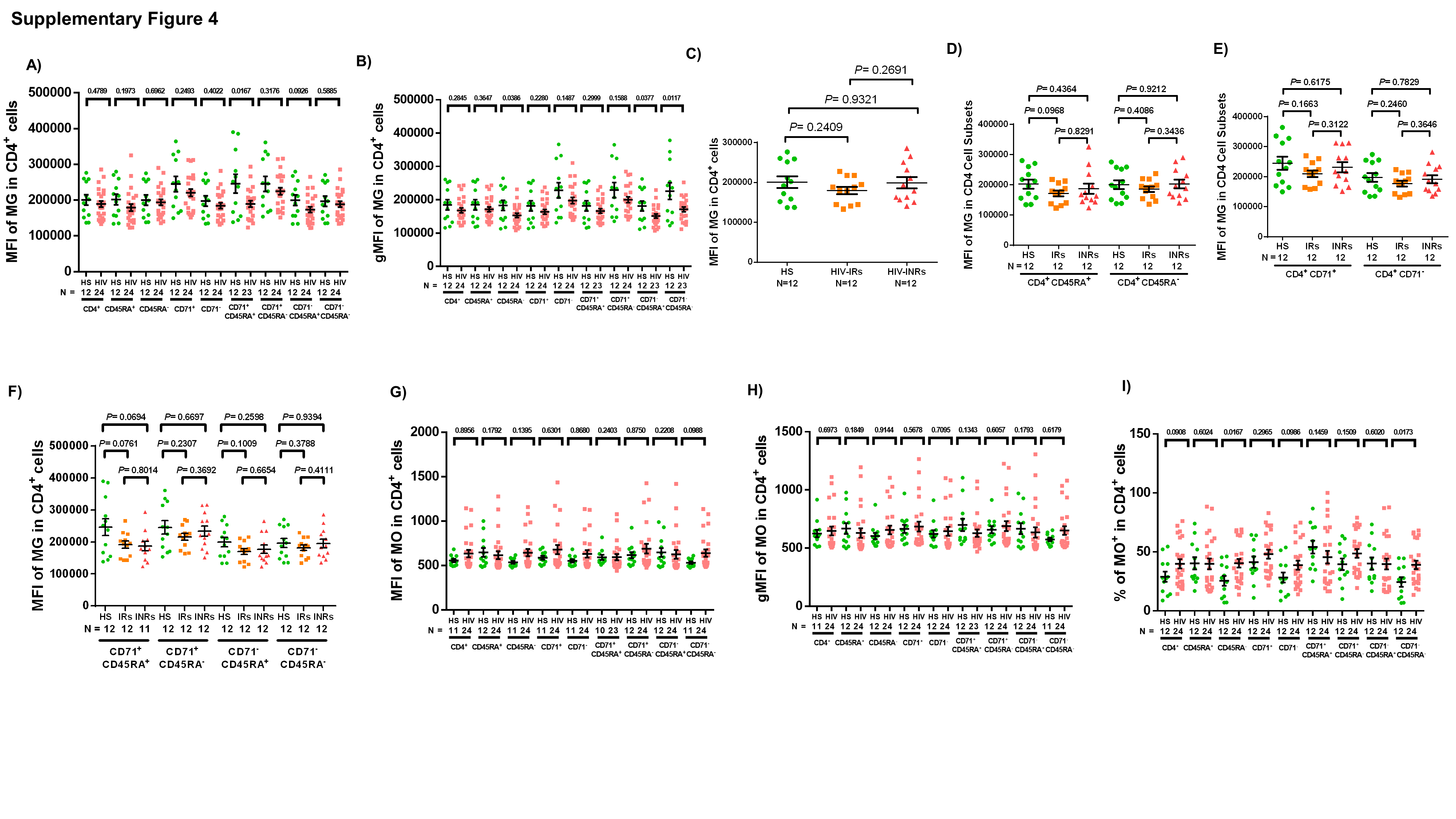

Supplement: Supplementary file 5 [file Image_4.tif]

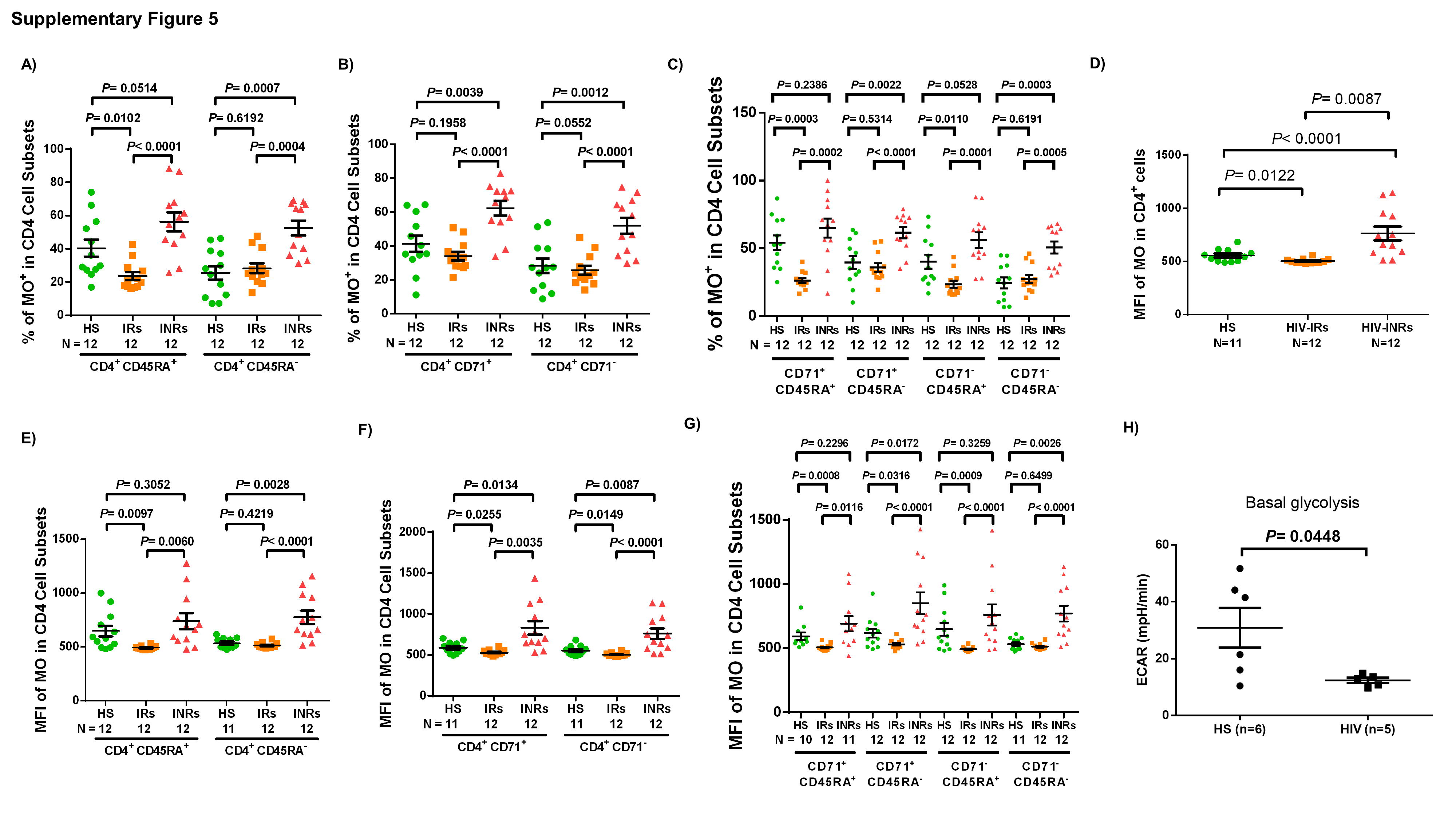

Supplement: Supplementary file 6 [file Image_5.tif]

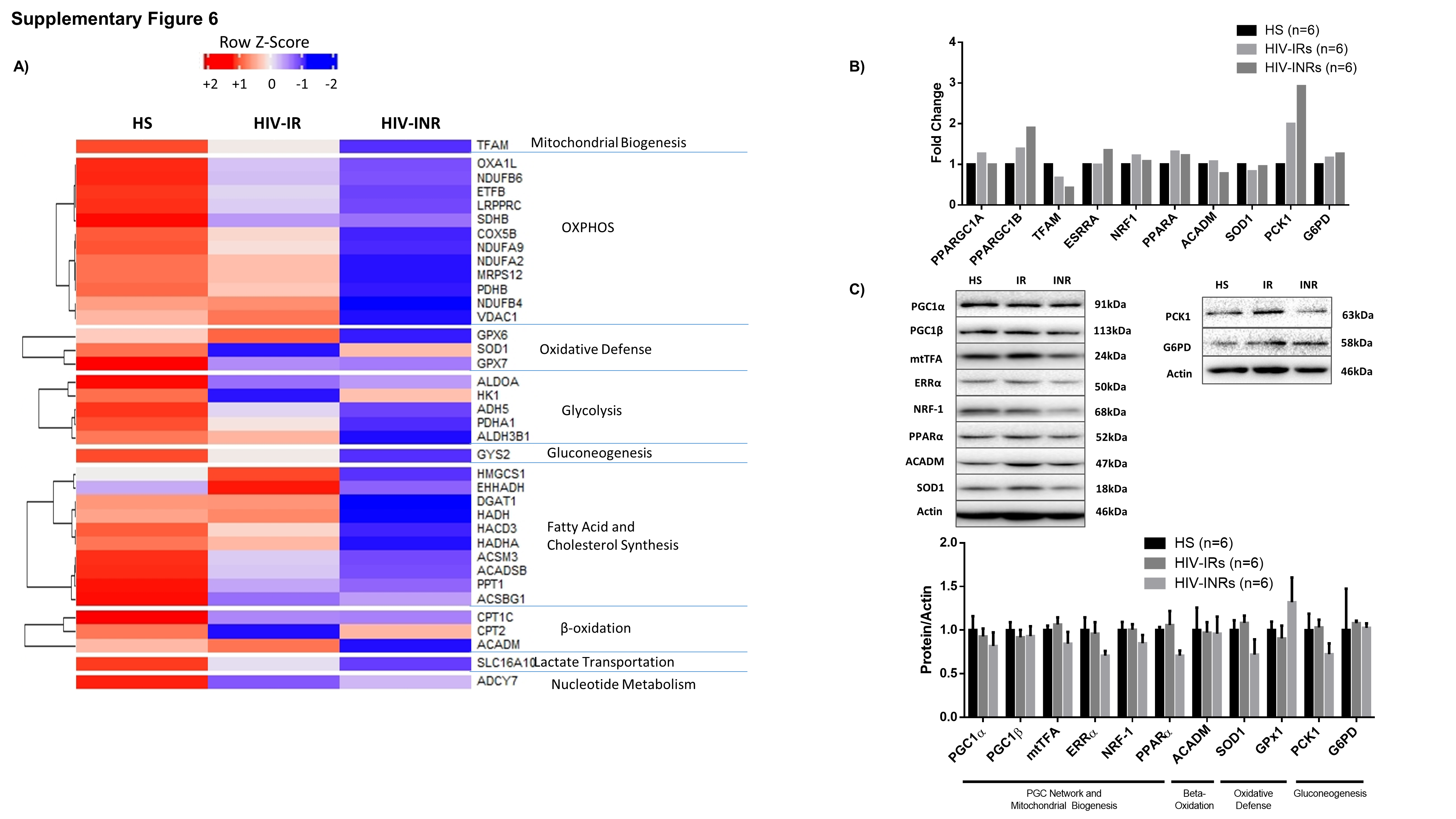

Supplement: Supplementary file 7 [file Image_6.tif]

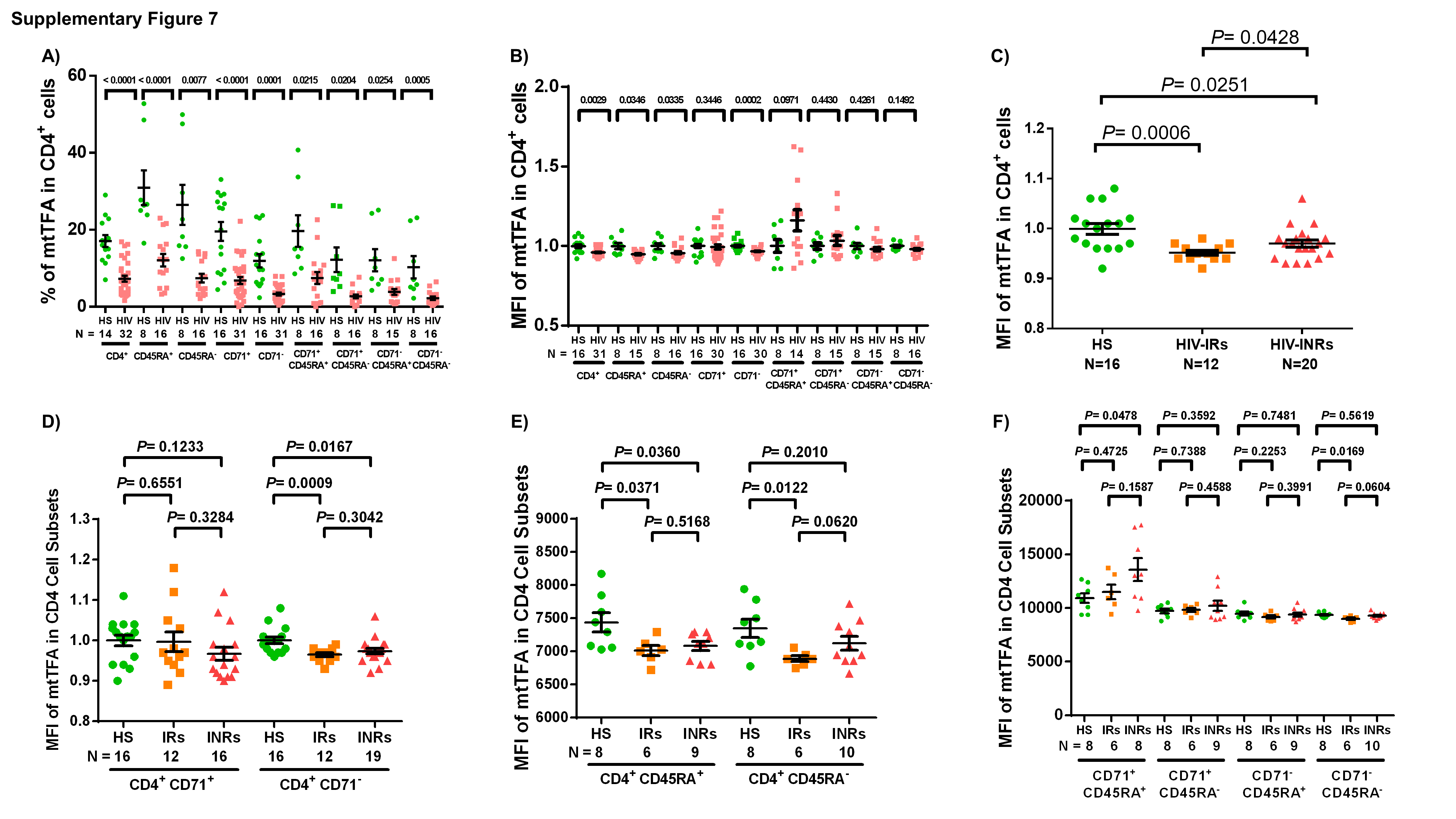

Supplement: Supplementary file 8 [file Image_7.tif]
